# Comparison of Regression and Machine Learning Methods in Depression Forecasting Among Home-Based Elderly Chinese: A Community Based Study

**DOI:** 10.3389/fpsyt.2021.764806

**Published:** 2022-01-17

**Authors:** Shaowu Lin, Yafei Wu, Ya Fang

**Affiliations:** ^1^The State Key Laboratory of Molecular Vaccine and Molecular Diagnostics, School of Public Health, Xiamen University, Xiamen, China; ^2^National Institute for Data Science in Health and Medicine, Xiamen University, Xiamen, China; ^3^Key Laboratory of Health Technology Assessment of Fujian Province, School of Public Health, Xiamen University, Xiamen, China

**Keywords:** machine learning, regression, depression, prediction, home-based elderly

## Abstract

**Background:**

Depression is highly prevalent and considered as the most common psychiatric disorder in home-based elderly, while study on forecasting depression risk in the elderly is still limited. In an endeavor to improve accuracy of depression forecasting, machine learning (ML) approaches have been recommended, in addition to the application of more traditional regression approaches.

**Methods:**

A prospective study was employed in home-based elderly Chinese, using baseline (2011) and follow-up (2013) data of the China Health and Retirement Longitudinal Study (CHARLS), a nationally representative cohort study. We compared four algorithms, including the regression-based models (logistic regression, lasso, ridge) and ML method (random forest). Model performance was assessed using repeated nested 10-fold cross-validation. As the main measure of predictive performance, we used the area under the receiver operating characteristic curve (AUC).

**Results:**

The mean AUCs of the four predictive models, logistic regression, lasso, ridge, and random forest, were 0.795, 0.794, 0.794, and 0.769, respectively. The main determinants were life satisfaction, self-reported memory, cognitive ability, ADL (activities of daily living) impairment, CESD-10 score. Life satisfaction increased the odds ratio of a future depression by 128.6% (logistic), 13.8% (lasso), and 13.2% (ridge), and cognitive ability was the most important predictor in random forest.

**Conclusions:**

The three regression-based models and one ML algorithm performed equally well in differentiating between a future depression case and a non-depression case in home-based elderly. When choosing a model, different considerations, however, such as easy operating, might in some instances lead to one model being prioritized over another.

## Introduction

Depression is the most common psychiatric disorder among elderly. A recent report conducted by WHO showed that 7% of older adults in the world suffered from depressive disorder (1). A report published in The Lancet estimated an additional 53.2 million cases of major depressive disorder globally (an increase of 27.6%) due to the COVID-19 pandemic (2). In addition, depression is usually associated with the elevated risk of other diseases (e.g., cardiac diseases) and mortality among the elderly people (3). In fact, the prevalence of geriatric depression was even worse in China, as research is usually focused on the developed areas (4). It is reasonable to assume that the onset of depression is different in low- and middle-income countries (LMIC) that present substantial economic disparity and low social support for the poorest people.

At present, some tools have been developed for depression risk prediction, while study on forecasting depression risk in the elderly is still limited. In an endeavor to improve accuracy of depression forecasting, machine learning (ML) approaches have been suggested, in addition to the application of more traditional regression approaches. Most studies reported ML models outperforming conventional models (5) owing to its advantage in handling the problem of “overfitting” (6–8).

However, to the best of our knowledge, the national prospective follow-up data is rarely used to study on the prediction of depression for home-based older adults (60 years old and above), and there is a lack of introduction of machine learning algorithms to solve the overfitting problem in the construction of geriatric depression prediction model. In fact, home-based eldercare will still be the primary means of eldercare in urban and rural communities in China for a long time in the future, due to the traditional Chinese “filial piety” thought. Furthermore, considerable evidence has accumulated that lifestyle (9), neuroimaging (10), biological (11), and environmental (12) factors resulting in depression. However, the presence of such risk factors could not always be employed to represent the presence of depression because these factors were obtained using group-level analysis (13, 14). ML is based on the individual-level analysis which enables to make full use of complex data set modeling and demonstrate strong predictive capabilities (15). So far most studies which the proposed ML methods have been mainly employed to screen the depressive risk of the community dwellers in cross-sectional design (16), or predicting treatment response in the clinical patients (17).

In this study, we explored the applicability of multiple models, using data from a large Chinese community based cohort to test the forecasting accuracy of four prediction methods, including three regression-based models and one machine learning model to get the optimal model in forecasting depression for the home-based elderly. This study has the potential to aid in mental health policies development. Additionally, our findings could provide an assistance for the development of risk identification tools in geriatric depression.

## Methods

### Data Sources

This study was based on nationwide data derived from the China Health and Retirement Longitudinal Study (CHARLS). It is an ongoing community-based cohort study among a nationally representative sample of Chinese adults aged 45 years and older, since 2011 and were followed up on every 2 years, with the latest wave of 2018 year, collecting comprehensive and detailed information on demographics, socioeconomic status, biomedical measurements, and health status and functioning. The detailed sampling design has been announced previously (18). The Biomedical Ethics Committee of Peking University approved this study, and all participants provided written informed consent. As the number of waves increases, the proportion of participants lost to follow-up is higher, and there are more missing predictive variables. Thus, our study selected valid samples of home-based elderly people aged 60 and above from the survey data in 2011 and 2013 to conduct research. Missing variables and non-home-based elders younger than 60 years old were excluded from the baseline. Participants with missing outcome variables in 2013 were also excluded.

#### Selection of Predictors

The stress-depression theory (19) deems that stress may cause or induce depression. Continuous stress from external stressors may directly lead to depression. Some researchers (20) put forward the hypothesis of stress sensitivity, suggesting that stress states such as chronic diseases and major injury events may increase the individual's susceptibility to depression. The cognitive theory of depression believes that life experiences at the beginning of childhood will act on individuals to form a stable cognitive structure or mental mode, which constitutes a framework for understanding oneself and the world. Among them, unfavorable life experiences and memories may form negative cognitive tendencies, resulting in long-lasting negative emotional states, and ultimately leading to depression (21). The theory of healthy social determination indicates that healthy social determinants are not only including the factors that directly cause disease, but also living and working environment determined by people's social status and resources. Among them, socioeconomic status is usually evaluated by three interrelated indicators of education, occupation and income (22).

In our study, 24 variables were included as candidate predictors. Specifically, predictors were firstly derived a priori from the forecasting study of depression in literature (23–25), as currently suggested for ML researches (26, 27). Second, we remained close to a suggested event per variable (EPV) value of 10, that is, to have 10 cases per predictor (7) in endeavor to avoid methodological disadvantages, such as picking unimportant predictor. And then a set of 24 variables with three categories in 2011 were selected as candidate predictors, including: (1) Demographic variables, such as geographical location, age, sex, rural/urban community, marital status. Geographical location was divided into eastern, central, and western regions according to the 2011 China health statistics yearbook. Marriage status was categorized as married (married/partnered), and single (never married/divorced/separated and widowed). (2) Socioeconomic variables were educational attainment, household per capita income, Huku status, occupational status, medical insurance (yes/no). Education attainment was allocated into two categories: low-level (elementary school and below) and high-level (middle school and above). Household per capita income was defined as total household income divided by number of people living in this household, and grouped into three categories based on the interval of 5,000 yuan. Huku status was categorized as agriculture, do not have and non-agriculture. Occupational status was divided into agricultural work, non-agricultural work, retired, and unemployed/never work. (3) Lifestyle and health status variables included cognitive ability, CESD-10 (Center for Epidemiologic Studies Depression Scale 10 items) score, sleeping time, self-reported memory, life satisfaction, ADL (activities of daily living) impairment, self-reported health status before 15 years old, social activities experience in the past month, smoking, drinking, chronic disease, disability, medical services experience, major misfortune injury experience. In accordance with previous studies (28, 29), cognitive ability was calculated using two categories: episodic memory and mental intactness. The word recall test was used to evaluate episodic memory. Specifically, examiners read a list of 10 random words, and participants were instructed to recall as many words as possible immediately afterward (immediate recall). The number of correctly recalled words was scored and indicated the participant's immediate recall. Ten minutes later, the participants were asked to recall the same list of words (delayed recall). Episodic memory scores were calculated as the average number of immediate and delayed word recalls and ranged from 0 to 10. The mental intactness based on some components of the mental status questions of the Telephone Interview of Cognitive Status (TICS) battery established to capture intactness or mental status of individuals. In CHARLS, mental status questions included serial subtraction of 7 from 100 (up to five times), the date (month, day, and year), the day of the week, the season of the year, and intersecting pentagon copying test. Answers to these questions are summed into a mental intactness score that ranges from 0 to 11. Global cognitive scores were calculated as the sum of the scores of episodic memory and mental intactness and ranged from 0 to 21. ADL impairment was measured by asking participants whether they had any difficulties taking a bath, eating, getting in and out of bed, dressing, using the toilet, defecating, doing housework, cooking, making phone calls, taking medicine, shopping, and managing finances due to health and memory problems during the past 3 months. The detailed evaluation methods were previously reported by Katz et al. (30) and Lawton and Brody (31). If any difficulty was reported and then the participant would be recognized as had difficulty in ADL. Social activities experience, including interacted with friends, done voluntary or charity work, stock investment, and other 8 kinds of social activity (done any of them in the past month/or not). Participants of the survey were asked whether they had been diagnosed with hypertension, dyslipidemia, diabetes or high blood glucose, cancer, chronic lung diseases, and other 9 kinds of chronic disease. If any disease was reported and then the participant would be recognized as had chronic disease. Medical services experience refers to a participant who have visited a hospital, or doctor's practice, or been visited by a doctor for outpatient care in the last month. Major misfortune injury experience refers to a participant who have ever been in a traffic accident or any other kind of major accidental injury.

### Outcome

The outcome was a self-reported depression in 2013 (binary: yes–no). Depression was evaluated using the 10-item Center for Epidemiologic Studies Depression Scale (CESD-10), the reliability and validity of this tool in Chinese elderly has been verified (32). The scores of the CESD-10 range from 0 to 30. The higher the score, the more severe the depression. Previous studies reported that a score of 10 on the CESD-10 had reliable levels of sensitivity (0.85) and specificity (0.80) in elderly Chinese (33). Thus, a respondent who had a CESD-10 score of at least 10 or was currently taking antidepressants was defined as suffering depression in our study.

### Model Construction and Evaluation

**Figure 1:**
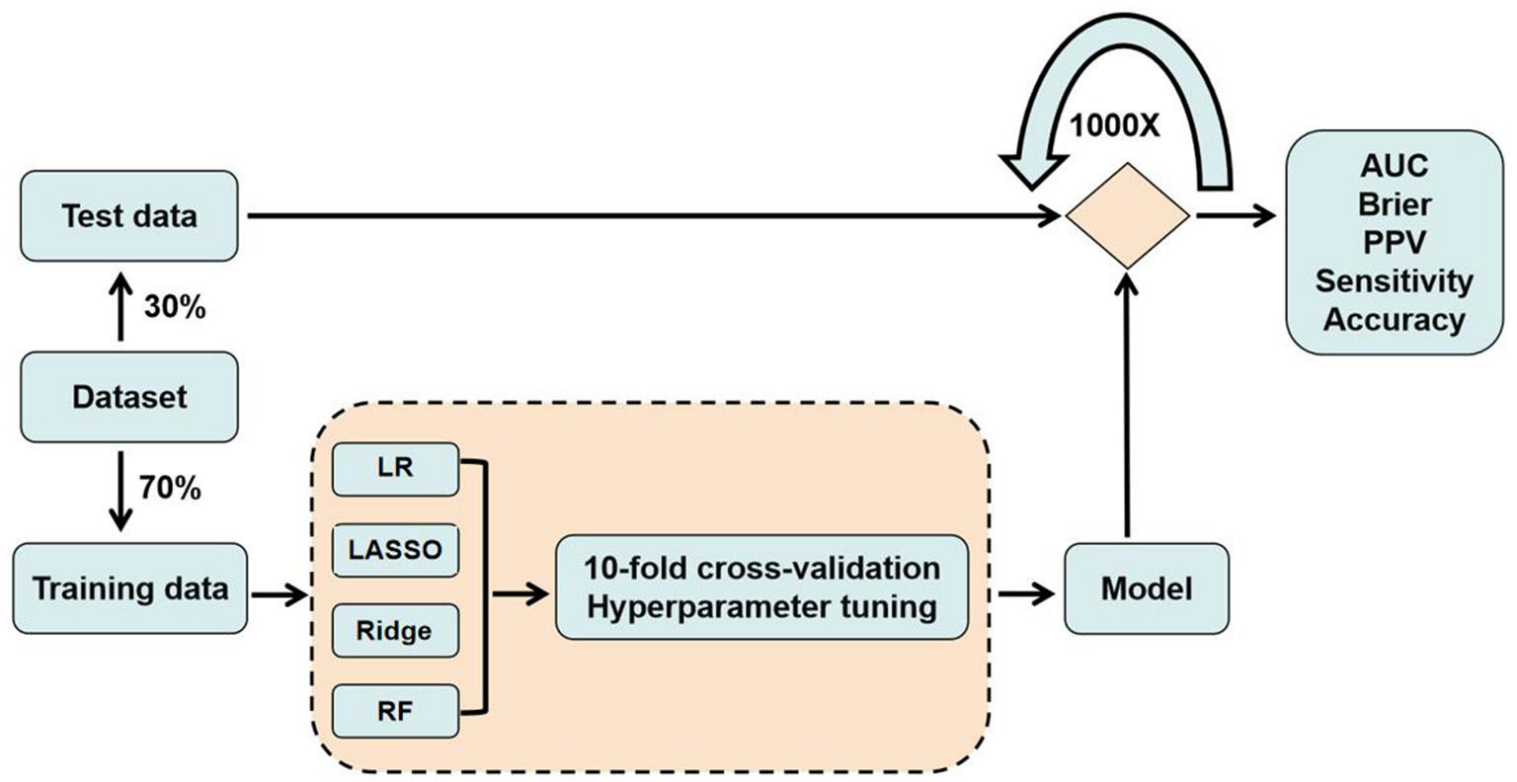
Model procedure for training and testing data.

Four prediction models were employed in our study: logistic regression, lasso (least absolute shrinkage and selection operator, lasso), ridge (both variants of logistic regression), and random forest, a widely used ML algorithm. All the four models are trained on the training set, and its prediction accuracy is tested on the test set. For each analysis, we randomly split our data into training (70% of the whole sample) and test datasets (30%). Moreover, in order to avoid performance estimates that are optimistically biased (overfitting), we applied repeated nested cross-validation, which is the suggested approach of verification (6). Repeated nested cross-validation hosts a two-stage process. At stage 1, the selected hyperparameters of the model were adjusted, such that the model's accuracy was optimized, as measured on a validation data set. Hyperparameters are distinct from the general model parameters (e.g., weights in a regression model) in that a configuration external to the model and its value cannot be estimated from the data. Adjusting hyperparameters means specifying how the model will learn from the data, for example, the extent of model complexity, for which we deployed a standard protocol with 10-fold cross-validation, hyperparameter tuning in the training dataset (Figure 1). We chose to adjust the parameter cost (cost of constraints violation) of the lasso and ridge regression models ranging from 0.001 to 0.3 (34). And in the random forest model, we selected the parameter of mtry (number of variables randomly sampled as candidates at each split) ranging from 1 to 23, while keeping the parameter ntree (number of trees to grow) at its initial value of 500, because adjusting this parameter is generally not suggested (35). At stage 2, the optimial parameter of stage 1 was used, with the purpose to acquire these models' final forecasting performance, for which the accuracy, sensitivity, precision and AUC of the discrimination index were selected to evaluate the prediction performance of the proposed model, using the test datasets. In addition, brief score was selected as the calibration index and all the testing process was repeated in 1,000 times to acquire a stable performance. For each classifier, we further calculated the relative importance of the predictors according to their contribution to prediction accuracy.

### Statistical Analysis

All the analyses were conducted on May 2, 2021. Continuous variables were represented by mean ± standard deviation under the condition of normal distribution, and skewed distribution were represented by median and interquartile range (IQR). Categorical variables were expressed as percentages. The *t*-test, Wilcoxon Mann-Whitney, and χ^2^ test were used to compare the statistical differences between the depressed group and the non-depressed group. SPSS 22 was used for the above analysis. The construction and evaluation of machine learning methods are completed by python 3.7 and Scikit-learn software toolkit. A bilateral *p* < 0.05 was considered statistically significant.

## Results

### Baseline Characteristics

**Figure 2:**
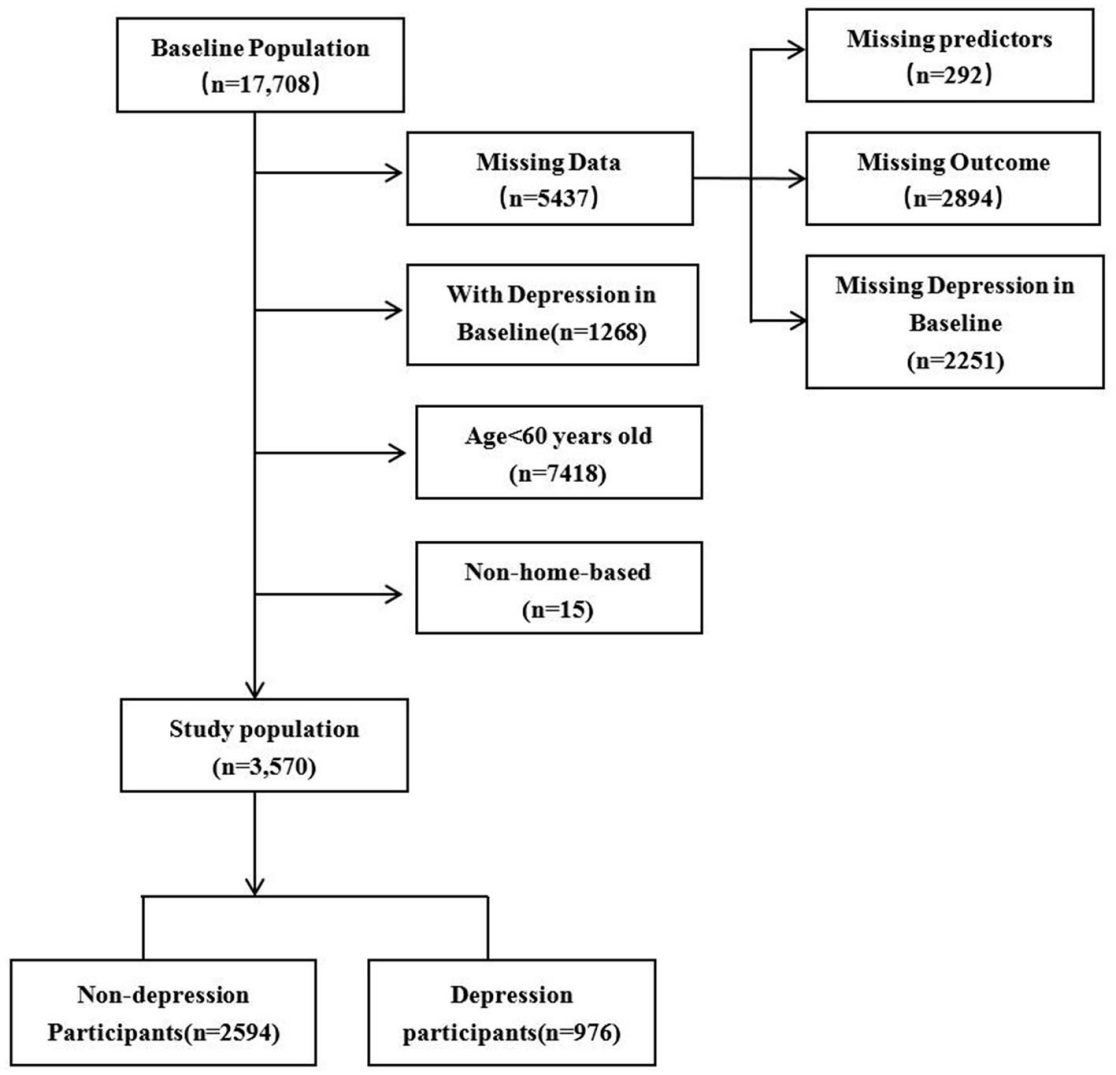
A flow chart for study population selection.

In our study, 17,708 participants were included in 2011. After excluding 5,437 participants with missing data (292 participants missing for predictors, 2,251 and 2,894 participants without depression status in 2011 and 2013, respectively), 1,268 participants with depression in 2011, 7,418 participants aged < 60 years, and 15 participants not in home-based care. Finally, 3,570 participants were included in our study. Among the follow-up participants, 976 were depression and 2,594 were non-depression participants in 2013, as shown in Figure 2. The prevalence of depression was 27.3% for the home-based elderly in 2013, of which the women present a much higher prevalence than men (56.56 vs. 43.44%). In the selection of predictors, we used *t*-test to analyze the differences between the depression group and the non-depression group, the age variables with insignificant differences (*P* > 0.05) were eliminated (Table 1), and the remaining 23 variables were included as predictors.

**Table 1:** Characteristics of the study population in 2011.

	**Depression (***n*** = 976)**	**Non-depression (***n*** = 2,594)**	***P*** **-value**
Age, years			
60-	659 (67.52%)	1,842 (71.01%)	0.059
70-	276 (28.28%)	674 (25.98%)	
80-	41 (4.20%)	78 (3.01%)	
Sex			
Male	424 (43.44%)	1,549(59.71%)	0.000
Female	552(56.56%)	1,045(40.29%)	
Rural/urban community			
Rural	708 (72.54%)	1,463 (56.40%)	0.000
Urban	268 (27.46%)	1,131 (43.60%)	
Hukou status			
Agriculture	835 (85.55%)	1,839 (70.89%)	0.000
Non-agriculture	141 (14.44%)	754 (29.11%)	
Geographic location			
East	278 (28.48%)	1,008 (38.86%)	0.000
Central	342 (35.04%)	877 (33.81%)	
West	356 (36.48%)	709 (27.33%)	
Marital status			
Single	223 (22.85%)	385 (14.84%)	0.000
Married	753 (77.15%)	2,209 (85.16%)	
Educational attainment			
Low	867 (88.83%)	1,922 (74.09%)	0.000
High	109 (11.17%)	672 (25.91%)	
Occupational status			
Agricultural work	514 (52.66%)	1,113 (42.91%)	0.000
Non-agricultural work	66 (6.76%)	348 (13.42%)	
Retired	379 (38.83%)	1,078 (41.56%)	
Unemployed/never work	17 (1.74%)	55 (2.12%)	
Household per capita income, yuan			
0-	714 (73.16%)	1,735 (66.89%)	0.001
5000-	131 (13.42%)	448 (17.27%)	
10000-	131 (13.42%)	411 (15.84%)	
Life satisfaction			
Satisfied	136 (13.93%)	724 (27.91%)	0.000
Medium	648 (66.39%)	1,718 (66.23%)	
Not satisfied	192 (19.67%)	152 (5.86%)	
Major misfortune injury experience			
Ever	124 (9.78%)	336 (9.41%)	0.007
Never	1,144 (90.22%)	3,234 (90.59%)	
Self-reported health status before 15 years old			
Good	689 (70.59%)	2,041 (78.68%)	0.000
Fair	188 (19.26%)	401 (15.46%)	
Poor	99 (10.14%)	152 (5.86%)	
Social activities			
Never	552 (56.56%)	1,219 (46.99%)	0.000
Ever	424 (43.44%)	1,375 (53.01%)	
Smoking			
Never	598 (61.27%)	1,375 (53.01%)	0.000
Ever	378 (38.73%)	1,219 (46.99%)	
Drinking			
Never	693 (71.00%)	1,672 (64.46%)	0.000
Ever	283 (29.00%)	922 (35.54%)	
Self-reported memory			
Good	77 (7.89%)	626 (24.13%)	0.000
Fair	364 (37.30%)	1,270 (48.96%)	
Poor	535 (54.82%)	698 (26.91%)	
Medical insurance			
No	66 (6.76%)	127 (4.90%)	0.028
Yes	910 (93.24%)	2,467 (95.10%)	
Medical service			
No	716 (73.36%)	2,163 (83.38%)	0.000
Yes	260 (26.64%)	431 (16.62%)	
Sleeping time, hour			
0-	301 (30.84%)	285 (10.99%)	0.000
4-	327 (33.50%)	898 (34.62%)	
6-	281 (28.79%)	1,168 (45.03%)	
8-	67 (6.86%)	243 (9.37%)	
Chronic disease			
No	181 (18.55%)	847 (32.65%)	0.000
Yes	795 (81.45%)	1,747 (67.35%)	
ADL impairment			
No	520 (53.28%)	2,023 (77.99%)	0.000
Yes	456 (46.72%)	571 (22.01%)	
Disability			
No	710 (72.75%)	2,174 (83.81%)	0.000
Yes	266 (27.25%)	420 (16.19%)	
Cognitive ability	7.51 ± 3.91	9.76 ± 3.99	0.000
CESD-10 score	5.73 ± 2.92	5.22 ± 2.80	0.000

### Predictive Performance Measures

**Figure 3:**
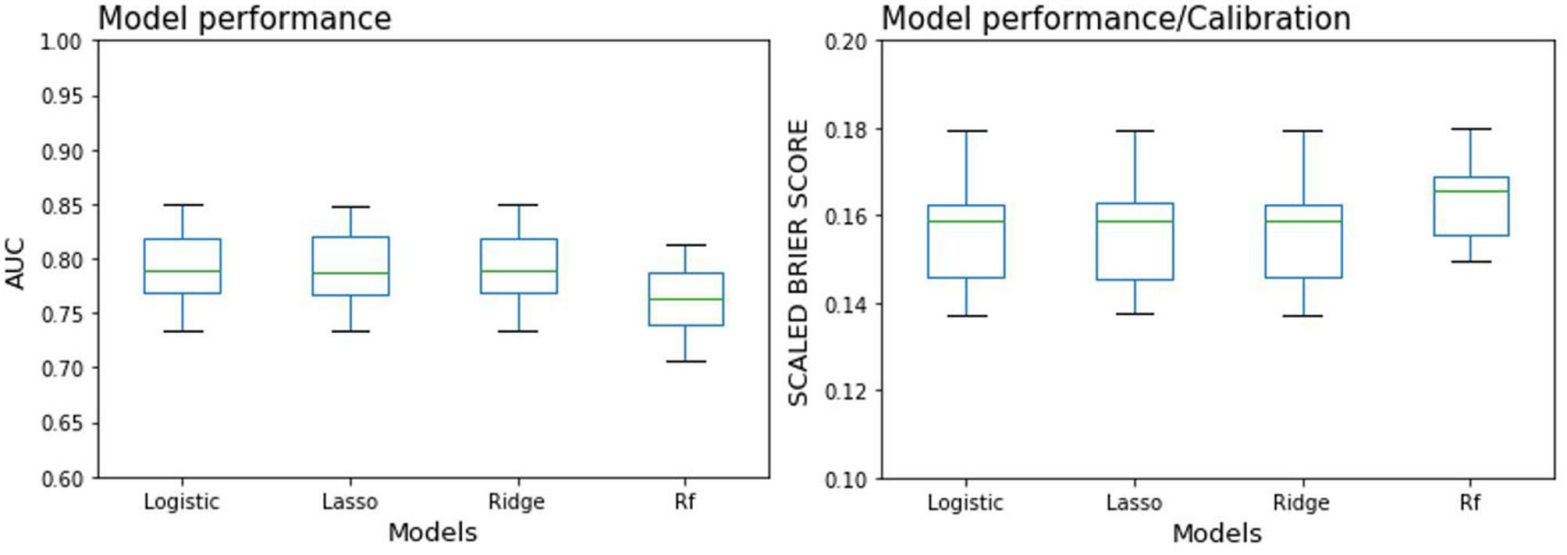
Boxplot of 100 resampling results for each prediction model (see median results in Table 2). Logistic, Logistic regression model; Rf, Random forest model. Left: Area under the curve (AUC). Right: Scaled Brier score, with values below zero indicating a model performance/calibration inferior to that of a chance prediction model applied to the validation dataset.

The values of AUC and scaled Brier score for all four models are shown in Table 2. AUC values were very similar amongst the four models for both mean (0.769–0.795) and median (0.771–0.788), with strongly overlapping boxplots (Figure 3), among which random forest is slightly lower than the other three. The scaled Brier score was highest for the random forest (mean: 0.164, median: 0.165) while the values of the other three models were all around 0.156 (mean) and ranged between 0.158 and 0.159 (median).

**Table 2:** Overview of the performance estimates for each prediction model.

**Model**	**AUC M**	**AUC Med**	**BS M**	**BS Med**	**Sens**	**PPV**
Logistic	0.795	0.788	0.156	0.159	0.404	0.656
Lasso	0.794	0.788	0.156	0.158	0.402	0.665
Ridge	0.794	0.788	0.156	0.159	0.402	0.654
Random forest	0.769	0.771	0.164	0.165	0.293	0.643

*AUC, area under the receiver operating curve; BS, Brier-scaled; Sens, sensitivity; PPV, positive predictive value; M, mean; Med, medium*.

### Sensitivity and Positive Predictive Value

Mean sensitivity and positive predictive value (PPV), each based on a predicted probability cutoff of 0.5, are shown in Table 2. Whereas, mean sensitivity ranged from 29.3% (RF) to 40.4% (logistic regression), both lasso and ridge regression showed similar sensitivities of around 40.2%. PPV amongst the four models fell into a close range between 64.3% (RF) and 66.5% (lasso), among them, the values of logistic and ridge were relatively close, being 0.656 and 0.654, respectively.

### Predictor Importance

**Figure 4:**
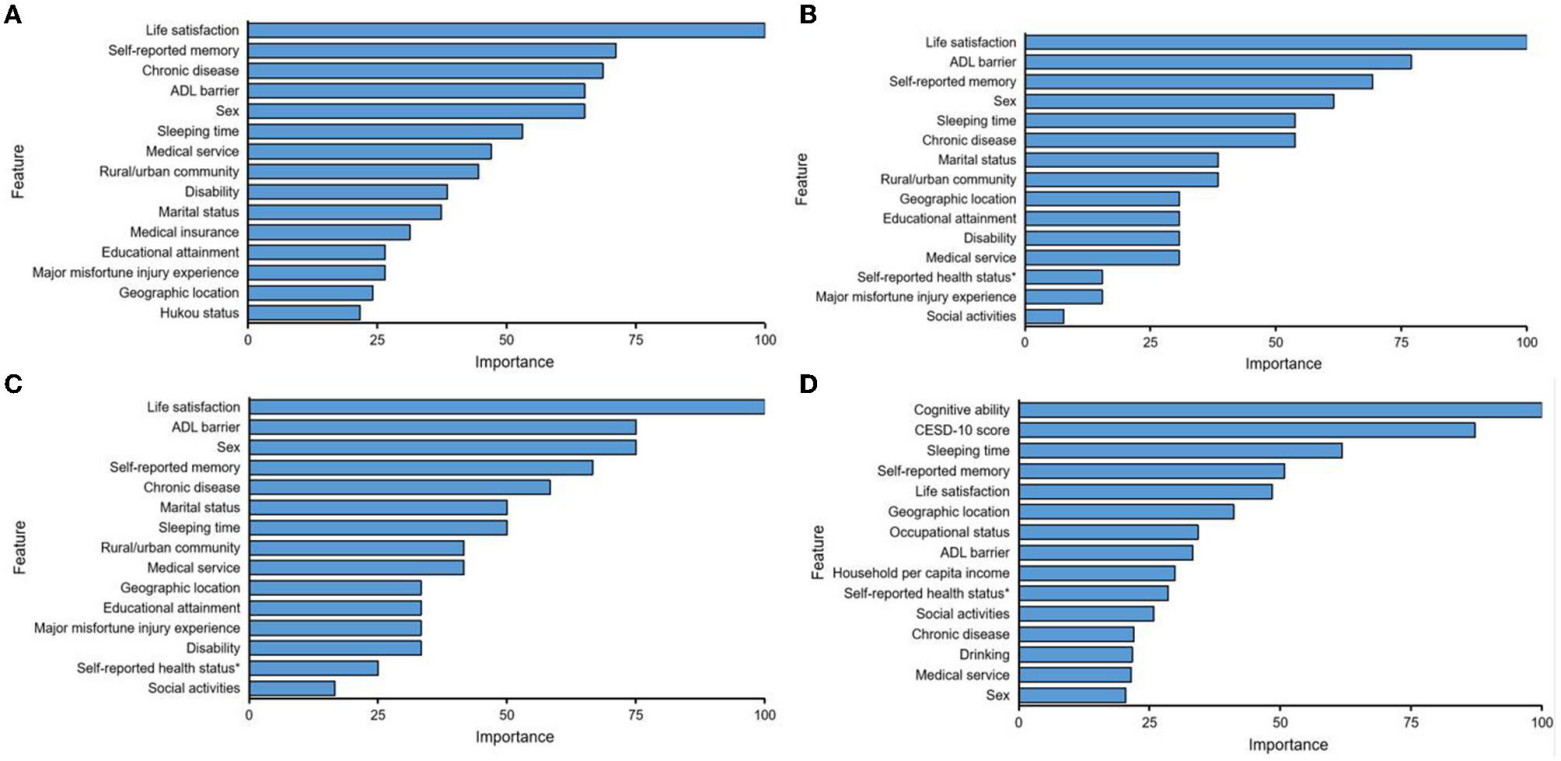
Display of the top 15 predictor variables for predicting depression by using Logistic **(A)**, Lasso **(B)**, Ridge **(C)**, and Random forest **(D)**. Feature: predictor; Overall: 100 = most important; and 0 = least important. Self-reported health status*: Self-reported health status before 15 years old.

Predictor importance for each model are summarized in Table 3 and Figure 4 displays the top 15 predictor variables ranked. The predictors all ranked in the top five among four prediction models were life satisfaction and self-reported memory. In the regression-based models, the most important predictor was life satisfaction, and it increased the odds of a future depression by 128.6% (logistic), 13.8% (lasso), and 13.2% (ridge), respectively. The most important predictor in random forest was cognitive ability. Whereas, self-reported memory ranked second in the logistic (79.9% risk increase), the ADL impairment ranked second in both lasso, and Ridge models (10.1 and 9.9% risk increase, respectively), and CESD-10 score ranked second in the random forest model. Chronic disease ranked third in logistic regression model, showing a risk increase for a future depression of around 77.0%. In the lasso model, the self-reported memory ranked third, with a risk increase of 9.0%. Whereas, sex ranked third in ridge model, sleeping time ranked third in random forest. Sex ranked fourth in both logistic and lasso regression, showing a risk increase for a future depression of around 72.2 and 8.7%, respectively. Self-reported memory ranked fourth in both ridge (8.5% risk increase) and random forest. Whereas, ADL impairment ranked fifth in logistic model (71.1% risk increase), chronic disease ranked fifth in both lasso and ridge, showing a risk increase for a future depression of around 7%. In random forest model, the fifth important predictor was life satisfaction. Regarding the overall predictor importance ranking, the lasso and ridge regression models represented a 52.2% concordance, that is, 12 of 23 predictors had the equivalent rank in both models. The concordance of rank ranged between 0 and 21.7% for all other possible comparisons of two models. When permitting ranks per predictor to differ by a maximum of 2 between two models, the concordance of ranks increased to 95.7% when comparing lasso and ridge regression. And also the range of concordant ranks increased to 8.7 and 65.2%. All three regression-based models delivered similarly high ranks to the predictors, including the sleeping time, medical services, urban/rural community, disability, marital status, respectively (ranking between sixth and tenth).

**Table 3:** Overview of the decreasing importance of the 23 baseline predictors for each prediction model.

**Predictive variables**	**Logistic β**	**OR**	Rank*	**%**	**Lasso β**	**OR**	Rank*	**%**	**Ridge β**	**OR**	Rank*	**%**	**Random forest importance**	Rank*
Life satisfaction	0.83	2.29	1	128.6	0.13	1.14	1	13.8	0.12	1.13	1	13.2	6.392	5
Self-reported memory	0.59	1.80	2	79.9	0.09	1.09	3	9.0	0.08	1.08	4	8.5	6.702	4
Chronic disease	0.57	1.77	3	77.0	0.07	1.07	5	6.9	0.07	1.07	5	7.4	2.911	12
Sex	0.54	1.72	4	72.2	0.08	1.09	4	8.7	0.09	1.09	3	9.3	2.704	15
ADL barrier	0.54	1.71	5	71.1	0.10	1.10	2	10.1	0.09	1.10	2	9.9	4.390	8
Sleeping time	−0.44	0.65	6	35.3	−0.07	0.94	6	6.4	−0.06	0.94	6	6.2	8.166	3
Medical service	0.39	1.47	7	46.9	0.04	1.04	10	4.3	0.05	1.05	9	5.0	2.834	14
Rural/urban community	−0.37	0.69	8	30.7	−0.05	0.95	8	5.0	−0.05	0.95	8	4.8	2.519	19
Disability	0.32	1.37	9	37.0	0.04	1.04	11	4.0	0.04	1.05	10	4.6	2.692	17
Marital status	−0.31	0.74	10	26.5	−0.05	0.95	7	5.0	−0.06	0.94	7	5.6	2.549	18
Medical insurance	−0.26	0.77	11	23.1	0.00	1.00	20	0.3	−0.02	0.98	15	2.3	1.254	23
Major misfortune injury experience	−0.22	0.80	12	20.0	−0.02	0.98	13	2.3	−0.04	0.96	13	3.5	1.749	22
Educational attainment	−0.22	0.80	13	19.8	−0.04	0.96	12	3.6	−0.04	0.96	12	3.9	1.891	21
Geographic location	0.20	1.22	14	21.8	0.04	1.04	9	4.3	0.04	1.04	11	4.4	5.426	6
Hukou status	−0.18	0.83	15	16.8	0.00	1.00	19	0.3	−0.01	0.99	18	1.1	1.895	20
Self-reported health status before 15 years old	0.16	1.17	16	16.8	0.02	1.02	14	2.3	0.03	1.03	14	2.8	3.771	10
Social activities	−0.10	0.91	17	9.5	−0.01	0.99	15	1.1	−0.02	0.98	16	1.8	3.407	11
Smoking	0.07	1.07	18	7.2	0.00	1.00	22	0.0	0.01	1.01	22	0.6	2.700	16
Household per capita income	−0.05	0.95	19	5.3	0.00	1.00	21	0.0	−0.01	0.99	21	0.6	3.948	9
CESD-10 score	0.04	1.04	20	4.3	0.01	1.01	18	0.5	0.01	1.01	20	0.6	11.508	2
Occupational status	−0.04	0.96	21	4.1	−0.01	0.99	16	1.1	−0.01	0.99	17	1.3	4.522	7
Cognitive ability	−0.04	0.96	22	4.0	−0.01	0.99	17	0.8	−0.01	0.99	19	0.8	13.206	1
Drinking	−0.01	1.00	23	0.5	0.00	1.00	23	0.0	0.01	1.01	23	0.5	2.865	13

Rank^*^ *, Order according to the predictor ranking of the logistic regression model; β, beta-coefficient of the (penalized) logistic regression model; OR, odds ratio; %, OR translated to percentage. The original importance values of the random forest model have been multiplied by 100, to avoid having to display too many digits*.

## Discussion

In our study, all four models of logistic, lasso, ridge regression and random forest represent comparable accuracy. According to AUC, our results (mean AUC ranging between 0.769 and 0.795) yield a superior prediction and have the ability to avoid over-fitting problems. In terms of Cohen's d, our AUC results can be translated to an effect size of about 1.1 (36), that is, our models have achieved the larger effects. When comparing the discriminative ability of our proposed models with other studies predicting depression on the individual level, our results fall into the upper level of the AUC range of the previous studies (0.65–0.84) (37). Moreover, the forecasting level is higher than previous studies with clinical samples, in which the AUC values ranged from 0.66 to 0.69 (38) and 0.63 to 0.76 (39), respectively. We suggest that the superiority of our proposed model over the studies of clinical individuals might have attributed to the heterogeneity and severity in the clinical sample. Indeed, they consist of highly heterogeneous participants in perspective of clinical (40), genetic variants and the factors of peripheral risk (41). Thus, once depression reached clinical diagnosis, it would be difficult to accurately forecast and intervene in effect. That is the point of which this study targets. By focusing on the prediction of future depression among national community sample, we could establish forecasting model with optimal performance measure and so as to detect the home-based elderly who have the risk to be depressed earlier, and prevent the condition of their illness from transforming into serious level and entering clinical treatment. In conclusion, we believe that our model can be used as basis for developing ML-based approaches in the prediction of short-term outcomes in LMICs, provided that the similarities of population structure and healthcare system patterns between these counties and China. Besides the clinical information and medical evaluations provided in LMICs, the prediction models can serve as a useful tool for setting priorities and guiding the allocation of limited resources. We also expect that our study might provide the incentive for other centers in LMICs to create similar community-based cohort, thus optimizing the prediction of clinical outcomes and improving the quality of care provided to patients with depression.

However, we forbear from comparisons with most of these studies, owing to the fundamental differences between such researches and our study, for example, in terms of sample type (mostly patients or certain area elderly vs. national community), sample size, study design (mostly cross-sectional vs. prospectively assessed data), data source (electronic health record data vs. national epidemiological data), and age group (almost exclusively adults vs. 60 years old and above). The only exception in terms of comparability is the North Korea study by Na et al. (42) who also used a representative national community sample to prospectively predict depression, and got an AUC value of 0.870 (random forest). However, the data they used consisted of a larger sample of 6,588 respondents (the study population is for all adults) with a CESD-11 and the 9 points of positive screening value. In our study, CESD-10 was used, with 10 points as the criterion for judging depression.

The question arises as to why most studies revealed that ML models outperforming conventional logistic or linear regression models, while some studies, including ours, represented comparable forecasting accuracy. Several evidences can be found in the reference, which report that the superiorities of ML depend on several data-related attributes, for example, on data amount, ML prefers “big data,” which is more suitable for solving high-dimensional complexity issues (e.g., non-linearity and high-order interactions), on predictor variables that consist of diverse data types and sources, and, moreover, on how troublesome group distinctive are to discover, which might be more difficult in two considerably homogeneous populations (e.g., depression with vs. major depressive disorder) than in heterogeneous populations (e.g., general community members with vs. depression). Another explication for comparable forecasting accuracy across the proposed models might be whether there is an adequate number of positive outcomes per predictor. Therefore, some studies report that ML (0.63–0.76) always outperform logistic or linear regression (0.62–0.70) (27, 43). However, even if the above conditions are met, Jin et al. found that the forecasting accuracy of logistic regression (AUC = 0.81) in the development of a prediction model for diabetic patients with depression comorbidities can also be comparable with machine learning such as multi-layer perceptron (AUC = 0.80) and random forest (AUC = 0.78) (44). Therefore, our results might not be completely interpreted by the above-mentioned standard that support the application of ML, which are not fully met by the CHARLS data. Of note, a current systematic review by Christodoulou et al. discovered no accuracy advantage of ML over logistic regression for clinical forecasting models in 71 studies across several medical research fields (e.g., psychiatry, cardiology, or oncology) (45). Similarly, Belsher et al. summarize that ML models currently are not ready for clinical applications across health systems concerning mental disorder (46), owing to several essential concerns that in their suggestion have remained unresolved (37).

In the discrimination of the model evaluation, we also calculated various metrics (sensitivity, PPV) in addition to the principal performance metric AUC. The combination of these metrics may yield a comprehensive effect in modeling evaluation, since each captures a particular aspect of model performance. Whereas, the AUC is suggested by some researchers as a global model performance metric [e.g., Bradley (47)], others recognize its extensive use (48), and yet others appeal it to be abandoned or substituted (49, 50). However, at present the AUC still seems to be active for comparing model accuracy across studies, which in our opinion is slightly less the study with sensitivity and the PPV. Unlike the AUC, sensitivity is not a global measure favorable across all probable thresholds of forecasting probabilities, while it is a topical measure for one given threshold. Specifically, sensitivity refers to the ability of identifying patients. The PPV refers to the proportion of all positive cases that are truly in ill, which depends on the incidence of diseases in specific populations, whereas the AUC does not (51). Therefore, PPV is difficult to be compared because of the inconsistent incidence of diseases in various populations. In addition, unlike AUC, the sensitivity of the four models are different. Among them, the three models of the logistic regression family performs in a close range, with sensitivities (0.396–0.403) and PPVs (0.65–0.67). By contrast, the sensitivity of random forest is lower (0.358), and the PPV value is around 0.642. Above all, the applicable condition of AUC and the other metrics such as sensitivity and PPV are very differently. One crucial aspect that must not be ignored is the situation in which one of these metrics is more suitable than another. In the comparison of different studies, AUC may be more suitable because it can capture the overall model performance. Whereas, it is necessary to set a specific probability threshold according to the specific situation to balance the modeling sensitivity and specificity in clinical applications. In the calibration of model evaluation, our study uses the Brier score for estimation. Unlike the AUC, the scaled Brier score does not differentiate with suggested cut-off ranks. We can therefore only descriptively note that the lasso regression performed the best in terms of the scaled Brier score (combination of prediction accuracy and calibration), whereas the other three models performed less well, with a 15.9–16.5% reduced scaled Brier score. Interestingly, even though the random forest model showed no particularly increased AUC values (see Figure 3, left panel), the scaled Brier score markedly differed from the other models, in terms of both the median and the variability (see Figure 3, right panel).

In our study, a difference in the rank distribution of predictors was observed between the random forest model and the regression models. Even within the logistic regression models there were some differences (see Table 3, e.g., logistic and lasso). The above results just reveal the issue of model applicability, that is, we may choose different predictors for different prediction methods. In addition, many ML models are known as black boxes (52), that is, even though ML can discover the rules from complex data sets and make accurate predictions, the self-learning approaches might have employed the predictors for calculating the outcome in such a way that humanities are not able to understand it, for example, 10th-order interaction. Regardless of this matter, the top five important predictors are coordance among the three regression models. Thanks to the random forest model, there are two predictors put in the top five ranks the same as the other three models. Cognitive ability, baseline depression score and sleeping time are the three other predictors ranked top five in the random forest model. Therefore, we could select the factors of life satisfaction, ADL impairment, self-reported memory, chronic disease, sex to predict depression when using regression methods, and the use of cognitive ability, CESD-10 score, sleeping time, life satisfaction, self-reported memory could help us acquire the optimal RF model in forecasting depression. It is interesting to note that life satisfaction is the most important predictor across all three regression models, and the random forest model also ranks it in the top 5, which verifying this predictor's value as supporting the highest forecasting power for a subsequent depression in home-based elderly. However, the comparability of predictors' rank among the three regression models needs to be further explored. In particular, we would stress that we compared the predictors' rank across models, so the size of the coefficients might not be compared between non-penalized and penalized logistic regression since the coefficients have been regularized (biased) in the latter one. The second most important predictor was ADL impairment in the lasso and ridge models. This represents the consistency of this predictor as being adventurous against depression, which is consistent with the findings of Jui-Hung Lin et al. (53). They found that compared with non-depressive patients, hospitalized depressed elderly showed more cognitive impairment and worse ability of daily living when admitted. However, a survey on the relationship between the occurrence of depression in the elderly Chinese Americans and functional dysfunction for more than 2 years revealed the inverse effect of depression on ADL, that is to say, the proportion of ADL and IADL disorders in the elderly with a higher level of depression is significantly higher than that of the same age group with a lower level of depression (54). In addition, some longitudinal studies have suggested that there was a bilateral relationship between depression and ADL disorders (55). Moreover, they will become more serious through interaction, which will cause greater damage to physical functions. In the logistic regression model, self-reported memory ranks second in importance, while self-reported memory ranks third in the lasso model, and fourth in both ridge and random forest. That is, the elderly with poorer memory are more likely to experience depression. Some studies have shown that the phenomenon of over-generalization of autobiographical memory is one of the susceptible factors for depression. This phenomenon may be an avoidance strategy adopted by depressed patients in order to avoid the painful emotional experience caused by activating related negative events when extracting specific memories (56). Some elderly people often recall negative events in the past and produce bad emotions such as regret and self-blame, but the memory of the good things in the past is beneficial to mental health. Because positive information can provide strong psychological support for the elderly and promote their pursuit of higher life goals (57). In the random forest model, the importance of cognitive ability ranks first. The cognitive impairment of the elderly may also be another susceptibility factor to depression. Therefore, cognitive intervention can be used to reduce the excessive generalized memory of depressed elderly people and promote them to maintain a good emotional state (58, 59). In addition, sex ranks third in importance in the ridge model, and ranks fourth and fifth in the lasso and logistic regression models, respectively. In logistic regression, the predictor of chronic disease ranks third, and ranks fifth in both lasso and ridge. It is worth mentioning that the baseline depression score and sleeping time rank second and third, respectively, in the random forest model. All in all, it is important to point out that the highest ranking features in the random forest model, such as cognitive ability, sleeping time, self-reported memory could share common underlying biological mechanisms, that is, the relationship between microglial priming (60) and the onset of depression. For the previous studies, it is a recognized fact that stress events could cause the development of depression, and clinical research evidence shows that microglia play a key role among them (61). At present, it is generally believed that microglia are in an inactive or resting state in a healthy brain, but are activated when the brain is inflamed or damaged. As early as in 2003, Tang (62) found that the microglia in the hippocampus of the brain showed an activated form after sleep deprivation was done in rats. Subsequently, a study by Mattei et al. (63) also found that the decline in memory level and cognitive ability could activate microglia. Due to the impact of cognitive impairment, memory declining and lack of sleep, the microglia in brain of older adults could be activated, and their morphology is shown to become larger and rounded, and secrete compounds that can instigate inflammation in the brain. Once the stimulation produced by peripheral and central inflammation seriously affects the functional-level behavioral process in the brain, it can lead to depression (64). However, the remaining predictors contribute less value for the depression forecasting such as the self-reported health status before 15 years old, which the period might be too far away to exert a greater influence on the onset of depression in elderly.

For clinical implications, the identified risk factors can be used to inform the community prescribers, e.g., using self-reported measures along with inexpensive cognitive testing for episodic memory and mental intactness, and to target preventive interventions for improving the remission of depression. Self-reported information including life satisfaction, ADL impairment, self-reported memory, history of chronic disease, sex, sleep time, CESD-10 score. Specially, such identified risk factors and training model could be used for developing risk assessment tool (e.g., risk calculator or APP system), which likely including four modules of “individual information input,” “data management,” “disease prediction,” and “intervention measures.” Once the index data of elderly has been entered into the system, the individual risk of depression can transmit to community prescribers, which can help them complete the decision-making processes earlier, and adopt lifestyle intervention or clinical treatment for patients as soon as possible.

We want to refer several advantages of our study. First, to the best of our knowledge this is the first study that applied ML procedures to prospectively predict depression in national home-based elderly (an assumption being supported by a current systematic review on the use of ML in the study of geriatric depression) (65). Second, we employed repeated nested cross-validation, which Krstajic et al. suggested as the best method for training and testing a forecasting model within a single dataset, that is, external validation being not available (6). Third, we complied with the reporting guidelines known as the TRIPOD statement (66). This advantage is also recommended by two systematic reviews (45, 67), who stricture the inconsistent reporting approaches of modeling accuracy across studies. Fourth, we used predictors that were a priori defined, taken from the depressive literature. We assume that this and the CHARLS data quality might have led to the very good (68) discriminative ability of the predictive models we applied.

There are also some issues to be further studied toward our current work. First, the forecasting performance of ML approaches such as random forest depends on the data amount, with larger amounts of data sometimes resulting in a higher performance result (69). In that aspect our data amount should be considered as a weak point. Some demographic factors only had a few categories due to limited data. While ML algorithms are good at capturing the non-linear and specific patterns contained in data. So this effect may be overcomed to a certain extent. However, most of the ML algorithms were suitable for prediction with big data, therefore, larger sample size are needed for a better prediction in our future research. It may also be argued that it is not data amount *per se* which matters, but rather the connection between predictor and outcome in the data, that is to say, whether additive or multiplicative (interaction). In the example of an additive association, ML algorithms such as random forest may thoroughly not be able to represent their forecasting potential, as reverse to a robust multiplicative connection. Second, we used self-reported data, which is inevitable in terms of several natural biases (e.g., recall bias). It also indicates that we lacked more detailed data other than self-report data, e.g., biological data. Third, the outcome variable depression in this research is evaluated by a self-rating scale, which might have led to an increased misclassification rate. Indeed, CESD-10 was not the clinical diagnosis tool, so it may hinder the clinical application of our prediction model, because we just constructed a CESD-10 based model due to the availability of data. However, we believe that our risk model could also provide guidance for the primary screening of high-risk populations in a community level. Also, we will try to develop risk prediction model based on clinically confirmed cases in future studies. Fourth, our study only employ nested-cross validation to evaluate the applicability of the model, as the distribution pattern of several variables might be different in other countries or areas, the results for China cannot be generalized, and thus it's necessary to carry out external validation among a wider population in the future. Fifth, the fact that the CESD-10 only evaluates the week before the assessment. Because of that, relevant information to the courses of the depression may be lost and even depression may not be accounted for, while for a more reliable determination of depression, more frequent evaluations and longer periods of follow-up are required. In our future research, we aim to verify our models in clinical condition and increase the sample size, using time series analysis method of ML, to improve the accuracy of predicting model.

## Conclusion

Our study revealed that ML models not always outperforming conventional regression models in the context of depression prediction. In conclusion, it may be possible to distinguish non- depression from those with depression in a community level. The decision support system based on the predictive models may be very valuable for community medical providers and could provide some reference for preliminary screening. Future work needs to focus on further improvement of predictive ability through advanced approaches and more discerning data, so as to facilitate better targeting of interventions to subgroups of patients at highest risk for adverse outcomes.

## Data Availability Statement

The original contributions presented in the study are included in the article/supplementary files, further inquiries can be directed to the corresponding author/s.

## Ethics Statement

The studies involving human participants were reviewed and approved by the Biomedical Ethics Committee of Peking University. The patients/participants provided their written informed consent to participate in this study. Written informed consent was obtained from the individual(s) for the publication of any potentially identifiable images or data included in this article.

## Author Contributions

SL, YW, and YF worked together on this article. SL conceived and designed the study. YW contributed to the data analysis. SL and YW drafted the manuscript. YF supervised and revised the article. All authors have approved the final article.

## Funding

This study was supported by the National Natural Science Foundation of China (No. 81973144) and the Field Investigation Foundation of Xiamen University (No. 2019GF032).

## Conflict of Interest

The authors declare that the research was conducted in the absence of any commercial or financial relationships that could be construed as a potential conflict of interest.

## Publisher's Note

All claims expressed in this article are solely those of the authors and do not necessarily represent those of their affiliated organizations, or those of the publisher, the editors and the reviewers. Any product that may be evaluated in this article, or claim that may be made by its manufacturer, is not guaranteed or endorsed by the publisher.
